# Accuracy of Four-Dimensional Computed Tomography and Different Imaging Modalities in Primary Hyperparathyroidism

**DOI:** 10.7759/cureus.50423

**Published:** 2023-12-13

**Authors:** Mazin Merdad, Ahmed M Mogharbel, Yousuf AlQurashi, Mohammed Nujoom, Mohammad Wazzan, Ahmed Abduljabbar, Razan K Daghistani, Shaza Samargandy, Amani Alhozali, Bander Alshehri, Nadia Batawil, Hani Z Marzouki

**Affiliations:** 1 Department of Otolaryngology, Head and Neck Surgery, King Abdulaziz University Hospital, Jeddah, SAU; 2 Department of Otolaryngology, Head and Neck Surgery, King Fahad Armed Forces Hospital, Jeddah, SAU; 3 Otolaryngology Head and Neck Surgery, Taif University, Taif, SAU; 4 Department of Radiology, King Abdulaziz University Hospital, Jeddah, SAU; 5 Department of Internal Medicine, King Abdulaziz University Hospital, Jeddah, SAU; 6 Department of Internal Medicine, King Abdulaziz University Faculty of Medicine, Jeddah, SAU; 7 Internal Medicine, University of Jeddah, Jeddah, SAU

**Keywords:** primary hyperparathyroidism, accuracy, ultrasonography, sestamibi scan, 4dct

## Abstract

Background

This study aimed to compare the accuracy of different imaging modalities in the preoperative localization of parathyroid pathology in primary hyperparathyroidism.

Methodology

This prospective study enrolled 70 patients who were biochemically diagnosed with primary hyperparathyroidism between 2021 and 2022 at our center. Patients underwent scanning using three imaging modalities, namely, Tc99m sestamibi scan (sestamibi), parathyroid ultrasonography, and four-dimensional computed tomography (4DCT). A descriptive analysis was performed to determine and compare the respective localizing sensitivities.

Results

The most common site of parathyroid adenoma (PA) was the left inferior parathyroid gland, seen in 28 (40%) patients. Three patients had false-positive imaging studies with no parathyroid pathology identified surgically or on histological examination. The median levels of parathyroid hormone decreased significantly (p < 0.001) after the surgery, with a median of 24.3 (1.90-121). Furthermore, 4DCT accomplished a sensitivity of 97.14% for diagnosing the side and 94.03% for overall localization of PA. This sensitivity was superior to the sensitivity of ultrasonography and sestamibi scan to detect the side and quadrant of the adenoma. 4DCT was significantly higher in sensitivity when compared to the combination of ultrasound and sestamibi (p < 0.001).

Conclusions

4DCT yielded the highest sensitivity in localizing parathyroid pathology from the imaging modalities studied with the lowest false-negative rate. Using ultrasound with 4DCT could be the most cost-effective combination for detecting primary hyperparathyroidism.

## Introduction

Primary hyperparathyroidism is a common endocrine disorder causing hypercalcemia and elevated parathyroid hormone (PTH) levels in ambulatory patients. The most common cause of this disorder is a single parathyroid adenoma (PA) in approximately 80% of cases, followed by four-gland hyperplasia (10-15%), multiple adenomas (5%), and, rarely, parathyroid neoplasms (>1%) [1-3].

The prevalence of primary hyperparathyroidism is 1-7 per 1,000 with an estimated incidence of 0.4-21.6 per 1,000. Studies have predicted primary hyperparathyroidism to be the leading cause of hypercalcemia worldwide [4].

Once a patient is diagnosed and fit for surgery, PA is managed through surgical excision or parathyroidectomy of one or more glands [5,6]. In the past, bilateral neck exploration (BNE) was considered the standard procedure when performing PA excisions. In BNE, all four parathyroid glands and both recurrent laryngeal nerves (RLNs) are exposed and clinically examined intraoperatively. More recently, imaging advancements have allowed for minimally invasive parathyroidectomy (MIP), which has provided better outcomes with shorter operations, lower surgical risk, reduced risk of airway compromise due to iatrogenic RLN injury, and fewer postoperative complications [7-10].

According to the literature, there is no optimal localization procedure as no single imaging modality has a satisfactory sensitivity, specificity, and positive predictive value (PPV) to prevent the need for other radiological studies [7]. The modalities used include Tc99m sestamibi scan (sestamibi), ultrasonography, four-dimensional computed tomography (4DCT), and positron emission tomography-computed tomography (PET-CT) [6].

The use of 4DCT for PA localization was first discussed in 2006. This imaging modality employs classical multiplanar reformations (sagittal, coronal, and axial), whereas the fourth dimension is the change in enhancement over time [11]. PAs show peak enhancement in the arterial phase, with washout from the arterial to the delayed phase and low attenuation in the non-contrast phase. Morphologically, central low-attenuation changes, polar vessel signs, and lobulated margins are all expected findings of PA observed on 4DCT [12].

In this study, we aim to compare the accuracy of different radiological modalities in localizing the cause of primary hyperparathyroidism preoperatively.

## Materials and methods

Study participants

We prospectively enrolled all 70 patients who were biochemically diagnosed with primary hyperparathyroidism who met one of the criteria for surgery [13] from 2021 to 2022. Patients who consented to participate in this study underwent scans with three localizing imaging modalities (4DCT, sestamibi scan, and ultrasonography) before undergoing parathyroid surgery. We excluded patients with non-primary causes of hyperparathyroidism and those who refused surgical intervention.

Data collection

Data were collected and subsequently categorized into the following four domains: (1) demographic data (age, sex, and nationality); (2) laboratory tests (preoperative, postoperative, post-excision, and latest PTH, calcium, and phosphate levels); (3) radiological findings (location of pathology and etiology in ultrasonography, sestamibi scan, and 4DCT); and (4) intraoperative and postoperative findings (location of pathology, surgery duration, date of surgery, histopathology result, and any complications during surgery).

The images were reviewed by experienced radiologists, who were blinded to the findings of the other modalities to eliminate reviewer bias. The findings were subsequently correlated with the intraoperative findings. The accuracy of true localization of the PA was confirmed by histopathological examination of the excised lesion and postoperative calcium and PTH findings.

Confidentiality and ethical approval

This study was ethically approved by the Institutional Review Board of King Abdulaziz University Hospital (reference number: 234-21). Only the principal investigator had access to the data. To ensure the privacy and confidentiality of participants, all identifying variables were removed. Informed consent was obtained from participants before participating in our study.

Statistical analyses

Continuous non-normally distributed data were reported using median and range and compared using the Wilcoxon signed-rank test for paired groups. Moreover, categorical variables were reported as numbers and percentages. The receiver operating characteristic (ROC) curve and area under the ROC curve (AUC) were used to assess the diagnostic accuracy of ultrasound, sestamibi scan, and 4DCT. The sensitivity, specificity, PPV, negative predictive value (NPV), positive likelihood ratio (+LR), and negative likelihood ratio (-LR) were calculated. All tests were considered significant at p-values <0.05. Statistical analysis was performed using SPSS software version 25 for Windows (IBM Corp., Armonk, NY, USA) and MedCalc software version 20 (Corporation 2017, Schoonjans 2018).

## Results

Patients’ demographic and tumor characteristics

Our study included 70 patients diagnosed with primary hyperparathyroidism, of whom 60 (85.71%) were females and 10 (14.3%) were males. The most common site was the left inferior gland followed by the right inferior gland (40% and 35.71%, respectively). In our sample, PA was confirmed in 61 (87.14%) patients, double parathyroid adenomas were found in three (4.28%) patients, two (2.85%) patients had parathyroid hyperplasia, one (1.42%) patient had a neoplastic parathyroid gland, and three (4.28%) patients were falsely localized and had no evidence of parathyroid pathology postoperatively (Table 1).

**Table 1:** Patient demographics and tumor characteristics. ** = median and range

Variables	Number (%)
Gender	
Female	60 (85.71%)
Male	10 (14.3%)
Location of parathyroid abnormality	
No pathology	3 (4.28%)
Left inferior	28 (40%)
Right inferior	25 (35.71%)
Right superior, right inferior, left superior, left inferior	2 (2.85%)
Left superior	5 (7.14%)
Right superior	4 (5.71%)
Left superior and left inferior	1 (1.42%)
Right inferior, left inferior	2 (2.85%)
Diameter of the parathyroid gland (mm)	14 (7–45)**
Histopathology results (postoperative results)	
Normal tissue	3 (4.28%)
Parathyroid adenoma	61 (87.14%)
Double adenoma	3 (4.28%)
Parathyroid hyperplasia	2 (2.85%)
Neoplastic parathyroid gland	1 (1.42%)

Outcomes of the surgery

The median levels of PTH decreased significantly (p < 0.001) after the surgery. The median PTH level was 24.3 (1.90-121) ng/L postoperatively, relative to 136 (26.07-2320) ng/L preoperatively. The median total calcium level was 2.65 (2-13) mg/dL and 2.39 (2-11) mg/dL pre- and postoperatively, respectively (p < 0.001) (Table 2).

**Table 2:** The difference between laboratory measurements before and after surgery. * = significant result

Variables	Median (range)	Wilcoxon signed-rank test	P-value
Parathyroid hormone (ng/L)			
Preoperative levels	136 (26.07–2320)	1.5	<0.001*
Postoperative levels	24.3 (1.90–121)		
Total calcium (mg/dL)			
Preoperative levels	2.65 (2–13)	9	<0.001*
Postoperative levels	2.39 (2–11)		
Ionized calcium (mg/dL)			
Preoperative levels	2.8 (2–14)	2	<0.001*
Postoperative levels	2.62 (2–12)		

Diagnostic accuracy of ultrasound, sestamibi, and 4DCT

The diagnostic accuracy of ultrasound was statistically significant (p < 0.001). The sensitivity and specificity of ultrasound were 49.25% and 100%, respectively, with an AUC of 0.746. The sensitivity and specificity of sestamibi were 71.64% and 33.33%, respectively (p = 0.883). Regarding the 4DCT, it accomplished a sensitivity of 94.03% and a specificity of 33.33% (p = 0.413). The pairwise comparison revealed no statistically significant diagnostic differences between ultrasound, sestamibi, and 4DCT in the diagnosis of PA.

Regarding the diagnosis of the pathology localization, ultrasound showed a statistically significant diagnostic accuracy (p < 0.001). This was accomplished with a sensitivity and specificity of 40% and 100%, respectively, with an AUC of 0.7. The sensitivity and specificity of sestamibi was 74.29% and 33.33%, respectively, with an AUC of 0.567. In this respect, 4DCT accomplished a sensitivity of 97.14% and a specificity of 33.33% (p = 0.958). The pairwise comparison revealed no statistically significant diagnostic differences between ultrasound, sestamibi, and 4DCT in the accurate detection of which side the lesion was on.

Ultrasonography had the highest false-negative rate of the imaging modalities studied, followed by sestamibi and 4DCT (48.57%, 27.14%, and 5.71%, respectively).

**Figure 1:**
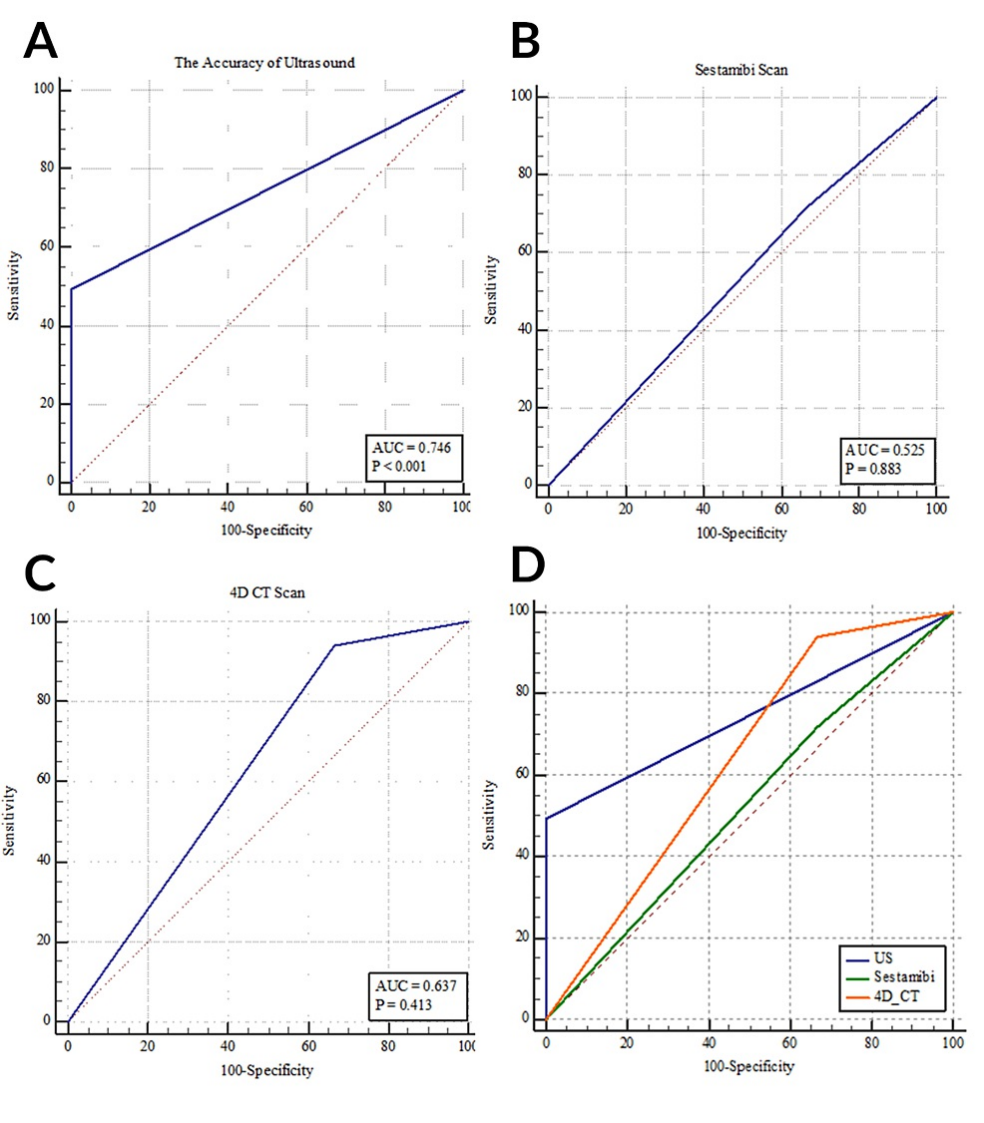
ROC showing the diagnostic accuracy of (a) ultrasound, (b) sestamibi, and (c) 4DCT in the overall diagnosis of parathyroid pathology. (d) ROC comparing the diagnostic accuracy of ultrasound, sestamibi, and 4DCT in the overall/quadrant localization of parathyroid pathology. 4DCT = four-dimensional computed tomography; ROC = receiver operating characteristics curve

**Figure 2:**
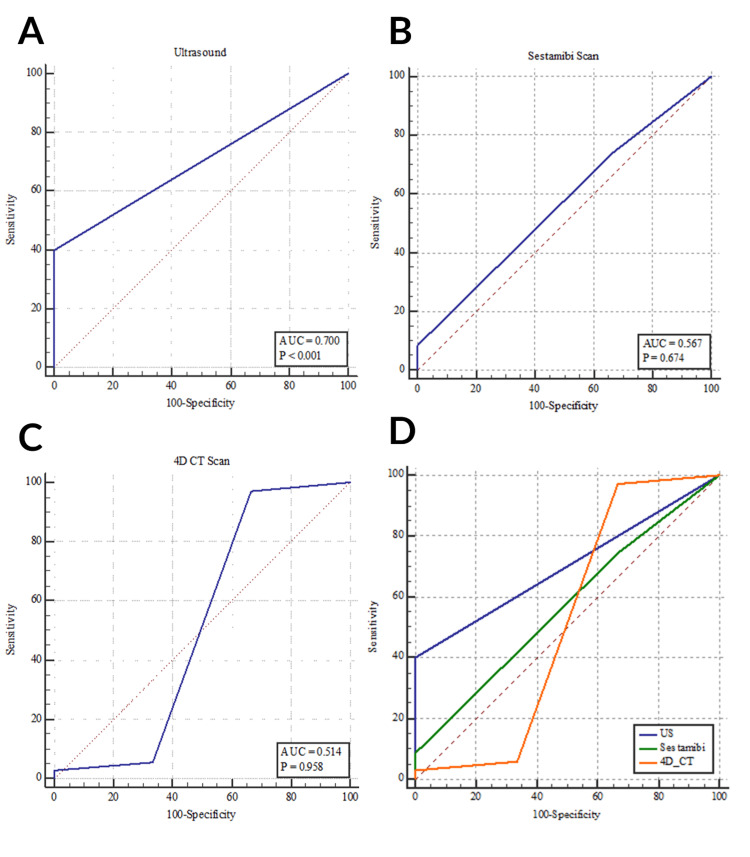
ROC showing the diagnostic accuracy of (a) ultrasound, (b) sestamibi, and (c) 4DCT in the diagnosis of the side of the parathyroid pathology. (d) ROC comparing the diagnostic accuracy of ultrasound, sestamibi, and 4DCT in the diagnosis of the side of the parathyroid pathology. 4DCT = four-dimensional computed tomography; ROC = receiver operating characteristics curve

The combination of ultrasound and sestamibi failed to localize pathology that was localized by 4DCT in 14.29% of our sample. Similarly, combining ultrasonography with 4DCT was negative while sestamibi was positive in 2.86% of our cases. All three imaging modalities were negative in 2.86% of patients (Tables 3-6; Figures 1, 2).

**Table 3:** Sensitivity of the different modalities versus surgery for the detection of the location and side of pathology. 4DCT = four-dimensional computed tomography; +LR = positive likelihood ratio; -LR = negative likelihood ratio; PPV = positive predictive value; NPV = negative predictive value; AUC = area under curve

Different modalities (right versus left)	Sensitivity	Specificity	+LR	-LR	PPV	NPV	AUC	P-value
Ultrasonography	40.00	100.00	-	0.60	100.0	12.5	0.700	<0.0001
Sestamibi scan	74.29	33.33	1.11	0.77	92.9	10.0	0.567	0.673
4DCT	97.14	33.33	1.46	0.086	94.4	50.0	0.514	0.958
Different modalities (quadrant localization)	Sensitivity	Specificity	+LR	-LR	PPV	NPV	AUC	P-value
Ultrasonography	49.25	100.00	0.5		100.0	8.1	0.746	<0.0001
Sestamibi scan	71.64	33.33	1.07	0.85	96.0	5	0.525	0.883
4DCT	94.03	33.33	1.41	0.18	96.9	20.0	0.637	0.413

**Table 4:** Comparison between the sensitivity of different modalities. 4DCT = four-dimensional computed tomography

Different modalities (right versus left)	Difference between areas	95% confidence interval	Z-test	P-value
Ultrasound versus sestamibi	0.133	-0.187 to 0.453	0.817	0.4142
Ultrasound versus 4DCT	0.186	-0.356 to 0.727	0.672	0.5017
Sestamibi versus 4DCT	0.0524	-0.760 to 0.865	0.126	0.8994
Different modalities (quadrant localization)	Difference between areas	95% confidence interval	Z-test	P-value
Ultrasound versus sestamibi	0.0774	-0.249 to 0.403	0.465	0.6417
Ultrasound versus 4DCT	0.0536	-0.550 to 0.657	0.174	0.8619
Sestamibi versus 4DCT	0.0238	-0.505 to 0.552	0.0883	0.9296

**Table 5:** Comparison between the different imaging modalities for false-negative rate. 4DCT = four-dimensional computed tomography

Modality	Rate
Ultrasound	34 (48.57%)
Sestamibi	19 (27.14%)
4DCT	4 (5.71%)

**Table 6:** False-negative rate analysis for combinations of modalities. 4DCT = four-dimensional computed tomography

Negative	Positive	Number
Ultrasound and sestamibi	4DCT	10 (14.29%)
Ultrasound and 4DCT	Sestamibi	2 (2.86%)
Ultrasound, sestamibi, and 4DCT	None	2 (2.86%)

Combining the sensitivities of diagnostic tools

The combination of sestamibi with 4DCT improved the diagnosis of PA in comparison to using sestamibi alone. The sensitivity was 98.31% in comparison to a sensitivity of 71.64% for sestamibi scan only (p < 0.001). In this respect, there was no significant difference between the combination of sestamibi and 4DCT (p = 0.68) when compared with 4DCT alone. The combination of ultrasonography and sestamibi obtained a sensitivity lower than that obtained by 4DCT (p < 0.001). The sensitivity of the former combination was 85.6%, relative to a sensitivity of 94.03% for 4DCT only. Combining the diagnostic results of ultrasound and 4DCT was significantly higher than the sensitivity of sestamibi only (p < 0.001) with a sensitivity of 96.97% and 71.64%, respectively (Tables 7-10).

**Table 7:** Comparison between the sensitivity of different modalities versus intraoperative findings. 4DCT = four-dimensional computed tomography

Different modalities	Sensitivity	Z-test	P-value
Sestamibi and 4DCT	98.31%	3.383	<0.001
Sestamibi	71.64%		

**Table 8:** Comparison between the sensitivity of different modalities versus intraoperative findings. 4DCT = four-dimensional computed tomography

Different modalities	Sensitivity	Z-test	P-value
Sestamibi and 4DCT	98.31%	1.48	0.68
4DCT	94.03%		

**Table 9:** Comparison between the sensitivity of different modalities versus intraoperative findings. 4DCT = four-dimensional computed tomography

Different modalities	Sensitivity	Z-test	P-value
Ultrasonography and sestamibi	85.6%	3.36	<0.001
4DCT	94.03%		

**Table 10:** Comparison between the sensitivity of different modalities versus intraoperative findings. 4DCT = four-dimensional computed tomography

Different modalities	Sensitivity	Z-test	P-value
Ultrasonography and 4DCT	96.97%	6.56	<0.001
Sestamibi	71.64%		

## Discussion

In our study, we found that 4DCT yielded the highest overall sensitivity in localizing hyperparathyroid pathology among the imaging modalities studied, with a significantly higher sensitivity when using 4DCT alone compared to the combination of sestamibi and ultrasonography. We also found that, despite the combination of 4DCT and sestamibi improving sensitivity in comparison to 4DCT alone, the improvement in sensitivity was not significant, which could imply that the addition of sestamibi scans to the diagnostic algorithm might be unnecessary.

We concluded that 4DCT was the most sensitive in localizing the overall location and the side of the PA, with the highest NPV. The higher sensitivities obtained with 4DCT are attributed to advancements in imaging by adding a fourth dimension (time) to the imaging [11]. These findings are significant in various aspects of healthcare, as improvements in imaging have led to smaller incisions, shorter surgery duration, and reduced postoperative complications [2]. These improvements, along with intraoperative PTH monitoring, are paramount as none of our patients experienced any complications, such as intraoperative major bleeding, postoperative infections, hungry bone syndrome, and reoperation. This is important because reoperation poses a great risk of morbidity among patients with persistent or recurrent primary hyperparathyroidism [14].

Multiple studies have investigated different preoperative imaging modalities in evaluating patients with primary hyperparathyroidism, with several agreeing on 4DCT being the most sensitive modality in detecting both single and multiglandular disease [6,7,14,15]. Although most studies discussed were retrospective, findings were consistent with our results, in which 4DCT was the most sensitive modality. This also applies to patients with a history of neck surgeries [14], which could affect the overall sensitivity of the modalities when compared to virgin necks, as in our study.

4DCT is valuable when other imaging modalities fail to localize the affected gland(s); in our sample, 10 patients had negative ultrasonography and sestamibi scans with a positive 4DCT. This correlates with the study by Day et al. who concluded that 4DCT was 89% sensitive in patients with negative modalities [16]. The addition of 4DCT for these patients changed their results from false negatives to true positives, which allowed us to localize the lesion and perform an MIP instead of a BNE, decreasing the overall surgical risk for these patients.

Future studies on the application of 4DCT and sestamibi scans should be performed to determine their effectiveness when applied to other tumors [17].

In the United States, the national average prices of ultrasonography, 4DCT, and sestamibi scans are $410, $712, and $1,479, respectively [18]. Wang et al. found that the combination of ultrasonography and 4DCT was more cost-effective than a sestamibi scan and that using more than one imaging modality is more cost-effective due to a reduced chance of bilateral surgical exploration [19]. The findings from Wang et al. are important as, based on our study, using 4DCT (with or without ultrasonography) is not only considered significantly more sensitive than the combination of a sestamibi scan and ultrasonography but also carries the additional benefit of reductions in cost to the patients. On average, the combination of ultrasonography and a sestamibi scan costs approximately two to three times as much as 4DCT with no added diagnostic value. This is in accordance with the guidelines published by the American Association of Endocrine Surgeons in managing primary hyperparathyroidism in 2016 [20], which state that the combination of ultrasonography with either 4DCT or sestamibi is the most cost-effective strategy.

Although operator-dependent, ultrasonography is an inexpensive and harmless modality with no risk of radiation exposure. Although the sensitivity of ultrasound for primary hyperparathyroidism is low, it is a very important diagnostic tool that could be employed as the first imaging modality to rule out other pathologies in the neck. Furthermore, ultrasonography is very accurate with a specificity nearing 100%. Positive ultrasonography is highly probable of a PA, but a negative result does not rule out pathology, and further imaging with 4DCT is warranted if the index of suspicion is high for better localization [2,7].

Strengths of this analysis include its prospective nature, which ensured that all our patients underwent all three of the imaging modalities discussed. This allowed us to directly compare the findings of the entire study group. We also included all patients with primary hyperparathyroidism irrespective of age, gender, or nationality. The blinding of the radiologists was also one of our strengths, which eliminated possible reviewer bias and allowed us to calculate the true sensitivities of each modality.

Our study is limited by the small cohort of participants, and further studies should be conducted with a larger sample size to provide more accurate results on this topic. Long-term outcomes for our patients were also not assessed and could be studied in future research.

## Conclusions

Based on our findings, 4DCT yielded the highest sensitivity in localizing parathyroid pathology from the imaging modalities studied with the lowest false-negative rate. Using ultrasound along with 4DCT could be the most cost-effective combination in detecting primary hyperparathyroidism, and may serve as a better modality compared to a sestamibi scan.
